# Sleep and energy drink consumption among Norwegian adolescents – a cross-sectional study

**DOI:** 10.1186/s12889-022-12972-w

**Published:** 2022-03-18

**Authors:** Siri Kaldenbach, Marja Leonhardt, Lars Lien, Asborg A. Bjærtnes, Tor A. Strand, Mads N. Holten-Andersen

**Affiliations:** 1 Department of Paediatric and Adolescent Medicine, Innlandet Hospital Trust, P.O. Box 104, Brumunddal, 2381 Norway; 2 Department of Clinical Medicine, University of Oslo, Oslo, Norway; 3 Norwegian National Advisory Unit on Concurrent Substance Abuse and Mental Health Disorders, Innlandet Hospital Trust, P.O. Box 104, Brumunddal, 2381 Norway; 4 Faculty for Health Studies, VID Specialized University, Oslo, Norway; 5 Faculty of Social and Health Sciences, Inland Norway University of Applied Sciences, Elverum, Norway; 6 Department of Research, Innlandet Hospital Trust, Lillehammer, Norway; 7 Center of International Health, Faculty of Medicine, University of Bergen, P.O. Box 7804, Bergen, 5020 Norway

**Keywords:** Energy drinks, Adolescents, Sleep, Shuteye latency, Population-based, Public health

## Abstract

**Background:**

Adolescents are recommended to get 8–10 h of sleep at night, yet more than 80% fail to obtain this goal. Energy drink (ED) consumption has been linked to later bedtime in adolescents. Therefore, we aimed to investigate the potential association between ED consumption and sleep duration, and shuteye latency among adolescents in Norway.

**Methods:**

This study was based on data from 15- to 16-year-old adolescents living in Oppland County in 2017. In total, 1353 adolescents were included in the analysis. Multiple regression models were used to estimate the associations between the frequency of ED consumption with sleep duration, shuteye latency, and getting 8 h of sleep.

**Results:**

Forty-six point five percent of the adolescents reported sleeping more than 8 h at night. Those who reported ED consumption at any frequency had significantly shorter sleep duration than those who did not. On average, high consumers of ED (consuming ED ≥ 4 times a week) had 0.95 (95% CI: 0.61, 1.28) hours (i.e., 57 min) less sleep than those who never consumed ED. In addition, high consumers had more than 25 min (95% CI: 13.95, 36.92) longer shuteye period than those who never consumed ED.

**Conclusion:**

Most ED consumers fail to obtain the recommended 8 h of sleep at night, which could be a consequence of shorter sleep duration and longer shuteye latency. We found a dose-response relationship between frequency of ED consumption and reduced sleep. Yet, the potential long-term effects of both ED consumption and insufficient sleep among adolescents remain unclear.

**Supplementary Information:**

The online version contains supplementary material available at 10.1186/s12889-022-12972-w.

Short sleep duration can result in poor mental and somatic health, and poor academic performance in adolescents [1, 2]. According to the sleep recommendation by the US National Sleep Foundation, adolescents aged 14–17 years should obtain 8–10 h of sleep each night [3]. However, in a recent study by Saxvig et al., 84.8% of Norwegian adolescents failed to obtain the recommended amount of sleep [4]. They found that the average sleep duration was 6:43 h on schooldays with a shuteye latency (the interval from bedtime to shuteye time) of 43 min.

Later bedtime and reduced sleep have been linked to screen time use before bedtime [5]. In a systematic review by Hale and Guan, more than 90% of studies included found a negative association between screen use and delayed bedtime and reduced total sleep duration [6]. In addition to overall screen time use, social media use, specifically night-time-specific social media use, has been associated with poorer sleep quality [5].

Poor sleep can result in anxiety and depression later in life. Sufficient sleep, and thereby proper restitution, is important for optimal neurocognitive and emotional functioning. In a study by Orchard et al., low total sleep time on school nights during adolescence predicted anxiety and depression later in life [7]. Physical activity is also an established factor in getting sufficient sleep. Research has shown that adolescents had lower levels of physical activity had shorter and poorer quality of sleep [8, 9].

Regular caffeine consumption has been associated with later bedtime in children and adolescents [10]. Caffeine is added to many different products including ED and is often referred to as a psychoactive drug with stimulating properties in the body, and has a half-life of about 4 h (ranging between 2 and 8 h) [11, 12]. According to the European Food Safety Authority (EFSA), adolescents have an increased chance of sleep latency and reduced sleep duration when consuming 1.4 mg/kg bodyweight of caffeine [12]. Indeed, the study by Mathew et al. showed that on the days that adolescents consumed caffeinated beverages they had a more variable sleep duration and timing of sleep [13].

Energy drink (ED) consumption has been linked to bedtime in adolescents, where more frequent consumption was associated with later bedtime [14, 15]. ED are non-alcoholic beverages containing at least 150 mg caffeine per litre, in addition to sugar, vitamins, minerals and amino acids [11, 16]. ED have gained increasing popularity in recent years and have been associated with higher intake of other sugary drinks and fast foods [17, 18].

Different patterns in ED consumption have been observed between boys and girls [19, 20]. Previous studies have identified that adolescents with high screen time [14], late bedtimes [15], shorter sleep duration [21] and drinking other sugar-sweetened beverages are more likely to consume ED more than once a week. ED have a high caffeine content which could contribute to later bedtime and shorter sleep duration.

Long shuteye latency among adolescents reduces their sleep opportunity, but what is keeping them from sleeping is unclear. The increasing trend in ED consumption [22] could contribute to poor sleep, as it is known that caffeine consumption can affect sleep quality, yet the link between ED consumption and sleep has not been well quantified. The main objective of this analysis was therefore to study the association between frequency of ED consumption on sleep duration and shuteye latency among adolescents in Norway. An additional aim was to investigate potential lifestyle related factors associated with sleep duration.

## Methods

### Design and setting

Data for the study was collected through a cross-sectional survey using a web-based questionnaire called the UngOpp survey. The current study was based on secondary analyses from the UngOpp survey. The survey was conducted in collaboration with the County Governor of Oppland, the supreme authority of all high schools in the county [23]. The survey included 70 questions on health, nutrition, and leisure activities, and perceived familial socio-economic status. The questionnaire was completed during school hours with the teacher present. All data was collected anonymously and was self-reported.

### Participants

10th grade students (15–16 years old) in all public schools in the district of Oppland, Norway, answered the questionnaire in April–May 2017 (*n* = 1793, 80.3% of those who were invited to participate). School attendance is mandatory until 10th grade in Norway. All 43 public schools (excluding three private schools, accounting for 24 students) in the former county of Oppland, Norway participated in the survey [24].

Only participants who provided complete data on ED consumption and items regarding sleep were included in the final analysis, which comprises 1353 participants (Fig. S1 in supplemental). Those giving implausible answers were removed from the analysis (*n* = 425, 23.7%).

### Ethical approval

All participation to the study was voluntary. The study was performed in agreement with the principles of the Declaration of Helsinki and the Health Research Act [25, 26]. Written informed consent was obtained by parents for students who were younger than 16 years and by the students themselves if they were 16 years old. The Regional Committee for Medical Research Ethics; Region South East, (University of Oslo), approved the study in 2017 (project number: 2016/1755).

### Outcome and independent variables

The main outcome measures were sleep duration and shuteye latency. Sleep was assessed using three questions: “When do you usually go to bed at night on schooldays?”, “When do you usually wake up on school days?”, and “how long does it usually take for you to fall asleep at night on school days?”. The participants were asked in the first two questions to specify the time at night they usually go to bed at night and the time they usually get out of bed in the morning. The third question assessed, in hours and minutes, the time it usually takes the participants to fall asleep. Based on these data, we could calculate time in bed (the interval from bedtime to getting up), shuteye latency (the interval from bedtime to sleep) and sleep duration/sleep period (time from shuteye to getting up).

According to recommendations of the US National Sleep Foundation, sleep duration on school days was set at a cut-off at 8 h where more than 8 h of sleep was categorized as “equal to or more than recommended” and below 8 h as “less than recommended” [3].

ED consumption was assessed with the question “How often do you usually drink energy drinks (Red Bull, Battery etc.)?” The participants could respond with one of seven incremental options, ranging from “never” to “several times a day”. The final two categories were combined into ED consumption ≥4 times a week because of a relative low percentage per answer option. This is also in line with previous classifications [20, 22].

### Description of baseline variables

All participants reported sex (male, female), size of community (below or above 20,000 inhabitants), and self-perceived financial situation of the family compared to other families with four options: poor family economy, medium family economy, good family economy and very good family economy [24]. For the purpose of the analysis, we combined the option good and very good family economy.

Leisure screen time was assessed using the question “On school days, how much time do you on average spend daily watching TV, movies, gaming or using social media?” with options on a four-point incremental scale ranging from “less than one hour” to “more than five hours”. The two lower options were merged into one response of “less than two hours” due to the relatively low number of respondents in these categories.

Physical activity was assessed by the question “How often do you perform physical activity which gets you out of breath or makes you sweaty?” with options on a six-point incremental scale ranging from “never” to “at least five times a week”.

### Statistical analysis

**Fig. 1:**
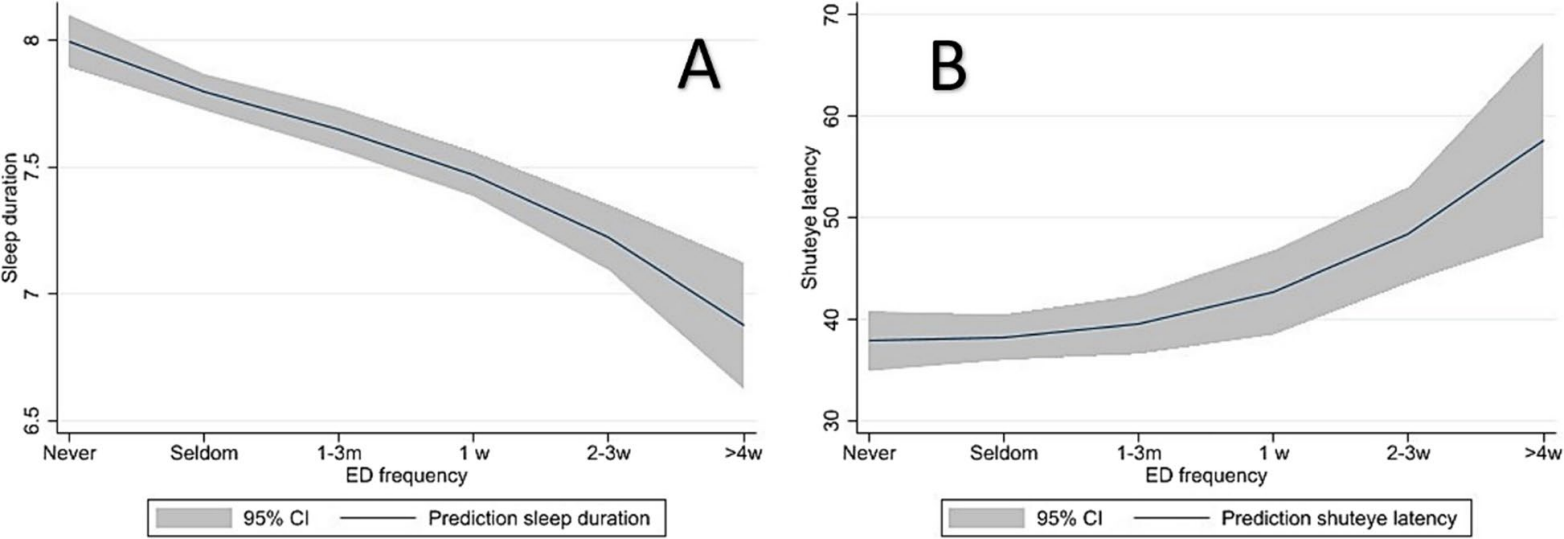
Sleep duration (**A**) and shuteye latency (**B**) according to frequency ofenergy drink consumption

The unadjusted associations between ED consumption and sleep duration (Fig. 1a), and between ED consumption and shuteye latency (Fig. 1b) were depicted using two-way fractional polynomials with confidence intervals (“twoway fpfitci” command in Stata). No other variables were included.

We identified predictors of sleep duration using a stepwise modelling approach. All variables, selected a-priori and shown in the baseline table (Table 1), were included in this process. Selection of predictors of ED consumption was based on previous analysis performed with the same type of datasets [20, 22]. Next, these predictors were combined and included as adjustment variables in multiple linear regression models that measured the association between ED consumption and sleep duration (in hours) and shuteye latency (minutes). Multicollinearity was assessed using variance inflation factor (VIF).

**Table 1:** Descriptive statistics of the background variables

	Total ***n*** = 1353	< 8 h sleep ***n*** = 724	≥8 h sleep ***n*** = 629
	*n* (%)	*n* (%)	*n* (%)
**Sex**			
Male	627 (46.3)	337 (46.5)	290 (46.1)
Female	726 (53.7)	387 (53.5)	339 (53.9)
**Perceived family economy**			
Poor family economy	44 (3.2)	25 (3.5)	19 (3.0)
Medium family economy	400 (29.6)	241 (33.3)	159 (25.3)
Good family economy	905 (66.9)	454 (62.6)	451 (71.7)
Missing	4 (0.3)	4 (0.6)	0
**Size of community**			
< 20,000 inhabitants	974 (72.0)	530 (73.2)	444 (70.6)
≥ 20,000 inhabitants	378 (27.9)	193 (26.7)	185 (29.4)
Missing	1 (0.1)	1 (0.1)	0
**Energy drink frequency**			
Never	408 (30.2)	176 (24.3)	232 (36.9)
Seldom	433 (32.0)	222 (30.7)	211 (33.6)
1–3 times a month	257 (19.0)	141 (19.5)	116 (18.4)
Once a week	112 (8.3)	77 (10.6)	35 (5.6)
2–3 times a week	97 (7.2)	72 (9.9)	25 (4.0)
≥ 4 times a week	46 (3.4)	36 (5.0)	10 (1.6)
**Physical activity frequency**			
Never	100 (7.4)	64 (8.8)	36 (5.7)
Once a week	201 (14.9)	108 (14.9)	93 (14.8)
2–3 times a week	447 (33.0)	239 (33.0)	208 (33.1)
4–6 times a week	456 (33.7)	248 (34.2)	208 (33.1)
Every day	147 (10.9)	63 (8.7)	84 (13.3)
Missing	2 (0.1)	3 (0.4)	0
**Leisure screen time weekdays**			
≤ 2 h	408 (30.2)	179 (24.7)	229 (36.4)
3–5 h	634 (46.9)	346 (47.8)	288 (5.8)
> 5 h	303 (22.4)	195 (26.9)	108 (17.2)
Missing	8 (0.6)	4 (0.6)	4 (0.6)

The associations between sufficient sleep (more than 8 h) and different categories of ED use and other independent variables were estimated in generalized linear models with the binomial distribution family and identity link function. These models yield risk differences (RD) and relative risk (RR).

STATA version 16.1 was used for all statistical analyses [27].

## Results

In total, 8.3% of the participants stated that they consumed ED once a week (Table 1), 7.2% consumed ED 2–3 times a week, and 3.4% consumed ED ≥ 4 times a week. 52.5% of the boys and 25.2% of the girls had consumed ED at any frequency. 22.4% of the adolescent reported more than 5 h screen time in their spare time during a weekday and at the same time, the majority of adolescents (77.6%) were physically active more than 2 times a week. More than half (53.5%) of the adolescents reported sleeping less than 8 h at night. Of these, 5.0% consumed ED ≥ 4 times a week, compared to 1.6% in the group who got more than 8 h of sleep at night. The average sleep duration was 7 h and 44 min (SD: 67.2 min) and the average shuteye latency (time it takes to fall asleep) was 40 min and 12 s (SD: 38 min).

Figure 1a shows the prediction of sleep duration based on ED consumption with confidence intervals. There seems to be dose-response relation between ED consumption and sleep. In the multiple linear regression model (Table 2), the frequency of ED consumption was negatively associated with length of sleep at night compared to those who never drank ED when adjusting for the other variables. This effect was smaller when adjusting for other variables. However, the effect of sex was larger in the adjusted analysis than in the unadjusted. Consuming ED as infrequent as 1–3 times a month predicted 0.48 h less sleep (CI: − 0.71, − 0.25) than those never drinking ED, while consuming ED ≥ 4 times a week predicted 0.95 h (CI: − 1.28, − 0.61) less sleep. Being physically active was positively associated with length of sleep.

**Table 2:** Energy drink consumption and sleep duration (in hours)

	Unadjusted		Adjusted	
	Coefficient	95% CI	Coefficient	95% CI
**Energy drink frequency**				
Never	Reference		Reference	
Seldom	−0.19	−0.34, −0.04	−0.14	−0.29, 0.00
1–3 times a month	−0.37	−0.54, −0.20	−0.32	−0.49, −0.15
Once a week	−0.50	−0.73, −0.27	−0.48	−0.71, −0.25
2–3 times a week	−0.77	−1.01, −0.53	−0.75	−0.99, −0.50
≥ 4 times a week	−1.13	−1.46, −0.80	−0.95	−1.28, −0.61
**Sex**				
Male	Reference		Reference	
Female	−0.06	−0.18, 0.06	−0.20	−0.32, −0.07
**Perceived family economy**				
Poor	Reference		Reference	
Medium	0.04	−0.31, 0.39	−0.12	− 0.45, 0.21
Good	0.22	−0.11, 0.56	0.01	−0.31, 0.34
**Physical activity**				
Never	Reference		Reference	
Once a week	0.31	0.04, 0.57	0.21	−0.05, 0.47
2–3 times a week	0.26	0.02, 0.50	0.10	−0.13, 0.33
4–6 times a week	0.39	0.15, 0.63	0.18	−0.05, 0.42
Every day	0.66	0.38, 0.94	0.36	0.08, 0.64
**Leisure screen time**				
≤ 2 h	Reference		Reference	
3–5 h	−0.26	−0.39, −0.12	−0.17	−0.30, −0.03
> 5 h	−0.71	−0.88, 0.55	−0.56	−0.73, −0.40

R^2^: 11.2%

There was an association between the frequency of ED consumption and longer shuteye latency when adjusting for other variables (Table 3). There were no indications of multicollinearity (VIF scores < 1.5 in the multiple linear models Adolescents who drank ED ≥ 4 times a week had 25.44 min (CI: 13.95, 36.92) longer shuteye latency compared to those who never drank ED. This was also shown in Fig. 1b, displaying the association between ED consumption and shuteye latency. Shuteye latency increased from approximately 38 min on average in adolescents not consuming ED to more than 60 min among those reporting to consume ED ≥ 4 times a week. However, adolescents who were physically active had a shorter shuteye latency compared to those who were never physically active.

**Table 3:** Energy drink consumption and shuteye latency (in minutes)

	Unadjusted		Adjusted	
	Coefficient	95% CI	Coefficient	95% CI
**Energy drink frequency**				
Never	Reference		Reference	
Seldom	1.06	−4.03, 6.16	0.25	−4.86, 5.36
1–3 times a month	4.05	−1.81, 9.92	3.72	−2.24, 9.68
Once a week	8.15	0.30, 16.0	9.90	1.94, 17.86
2–3 times a week	4.50	−3.77, 12.77	5.77	−2.75, 14.29
≥ 4 times a week	25.54	14.33, 36.74	25.44	13.95, 36.92
**Sex**				
Male	Reference		Reference	
Female	5.14	1.11, 9.17	6.63	2.35, 10.90
**Perceived family economy**				
Poor	Reference		Reference	
Medium	0.23	−11.47, 11.93	3.53	−8.07, 15.14
Good	−5.11	−16.48, 6.26	0.29	− 11.07, 11.65
**Physical activity**				
Never	Reference		Reference	
Once a week	−9.48	−18.34, −0.62	−7.77	− 16.67, 1.12
2–3 times a week	−11.28	−19.27, −3.28	−9.17	−17.25, −1.09
4–6 times a week	−17.87	−25.85, −9.90	−14.85	−22.99, −6.71
Every day	−23.05	−32.44, −13.67	−17.94	−27.52, −8.36
**Leisure screen time**				
≤ 2 h	Reference		Reference	
3–5 h	5.67	1.00, 10.35	3.02	−1.68, 7.73
> 5 h	10.94	5.37, 16.5	5.76	0.03, 11.50

R^2^: 5.1%

Table 4 shows the associations (RD and RR) between categories of ED consumers and sleep: A lower proportion of those who consumed ED more than once a month obtained at least 8 h of sleep than the reference group (never consumed ED). For example, the RD between adolescents never consuming ED and adolescents consuming ED ≥ 4 times a week was − 0.31 (CI: − 0.43, − 0.19) meaning that adolescent high ED consumers had a 31-percentage point less likelihood of obtaining 8 h of sleep when adjusting for the other variables in the analysis. In addition, adolescents who had more than 5 h leisure screen time had a RD of − 0.15 (CI: − 0.23, − 0.08) meaning that they had 15-percentage point less likelihood of obtaining the recommended 8 h of sleep compared to those who did get the recommended amount. On the other hand, adolescents who reported daily physical activity had a RD of 0.13 (0.01, 0.25) which means that these adolescents had a 13-percentage point increased likelihood of getting more than 8 h of sleep at night.

**Table 4:** The adjusted association between energy drink consumption and getting more than 8 h sleep

	Risk differences	95% CI	Relative Risk	95% CI
**Energy drink frequency**				
Never	Reference		Reference	
Seldom	−0.07	−0.13, 0.01	0.89	0.78, 1.01
1–3 times a month	−0.10	−0.18, −0.02	0.83	0.71, 0.97
Once a week	−0.24	−0.34, −0.14	0.57	0.43, 0.76
2–3 times a week	−0.31	−0.40, −0.21	0.47	0.33, 0.67
≥ 4 times a week	−0.31	−0.43, −0.19	0.43	0.25, 0.75
**Sex**				
Male	Reference		Reference	
Female	−0.05	−0.10, 0.00	0.90	0.80, 1.01
**Perceived family economy**				
Poor	Reference			
Medium	−0.11	−0.25, 0.04	0.87	0.61, 1.25
Good	−0.02	−0.16, 0.13	1.06	0.75, 1.50
**Physical activity**				
Never	Reference		Reference	
Once a week	0.09	−0.02, 0.19	1.15	0.87, 1.54
2–3 times a week	0.07	−0.02, 0.16	1.13	0.86, 1.48
4–6 times a week	0.05	−0.04, 0.15	1.07	0.82, 1.41
Every day	0.13	0.01, 0.25	1.24	0.93, 1.66
**Leisure screen time**				
≤ 2 h	Reference		Reference	
3–5 h	−0.07	−0.13, −0.01	0.88	0.78, 0.99
> 5 h	−0.15	−0.23, −0.08	0.72	0.61, 0.86

## Discussion

This is the first study to quantify sleep duration and shuteye latency in relation to ED consumption among adolescents. Our study showed that ED consumption was negatively associated with sleep at night and positively associated to the shuteye latency. These associations were not substantially affected by adjusting for sex, perceived family economy, physical activity and leisure screen-time. In addition, adolescents who consumed ED once a week or more had more than 20 percentage points higher risk of not getting the recommended amount of sleep at night. ED consumption seemingly adds to the burden of less sleep in a critical developmental stage when the adolescents need sufficient sleep to perform well in school and other social activities.

The association between frequency of ED consumption and both shorter sleep duration and longer shuteye period is evident according to our study. This is in line with previous studies showing an association between ED consumption and various sleeping problems, especially sleep duration [18, 21, 28]. In addition, “to get more energy” has been reported as an important reason for consuming ED. [29] Based on this, one could hypothesize that adolescents start drinking ED because they are sleepy and need energy. This could result in getting less sleep with accompanying tiredness the next day, even though we do not know at what time of day the adolescents consume ED. A vicious circle could accordingly be established where ED consumption leads to less sleep which then again entails increasing ED consumption resulting from tiredness. This is plausible and in line with other studies when considering the caffeine content in ED and its known effects on sleep [10, 13]. In addition, the association found in our study suggests a dose-response relationship. Yet, the direction of the association and causality cannot be determined based on the design of the current study.

Previous studies found a relationship between ED consumption and health problems that was mediated through going to bed late [29]. The longer shuteye period found in our study was also mediated by increased leisure screen-time. Previous studies have found that electronic media use in bed resulted in later bedtime and shorter sleep duration [5]. The combination of ED and screen-time could potentiate the adverse effects on sleep such as shorter sleep duration and longer shuteye period. However, in the current study, neither time of day when the adolescents used electronic devices not when they consumed ED were not recorded. Adolescents are exposed to many different types of advertisement through social media. In the study by Buchanen et al., adolescents who were exposed to digital marketing of ED were more likely to consume ED. [30] In other words, our observation of an association between ED use and sleep could in part be explained by reverse causality. From this, one could think that going to bed late or having a longer shuteye period whether it is because of ED consumption or because of electronic media usage, the exposure of digital marketing on ED together with tiredness can affect the adolescent to consume ED the next day. However, more detailed information on timing of screen-time and ED consumption among adolescents are currently lacking.

Our results suggest that ED consumption at any frequency increases the risk that adolescents do not obtain the recommended 8 h of sleep. In our study, 18.9% of the adolescents had consumed ED more than once a week. This is in line with previous studies with similar age groups [14, 20, 31]. The association between ED consumption and hours of sleep was found even though the timing of ED consumption was not known. According to our findings, 53.5% failed to obtain the recommended > 8 h of sleep which is less than the 84.8% found by Saxvig et al. [4]. Insufficient sleep is related to poor school performance [32] and might lead to poor mental and somatic health. We also found that physical activity increased the likelihood of getting sufficient sleep, which again is related to somatic health. There might also be other factors associated with sleep and health, which could be in the causal pathway between these variables. Adolescence is an important developmental period in life and behaviour during this period may affect life-long health and health related habits [33]. Therefore, raising awareness about the negative health effects of ED consumption among adolescents is needed.

### Strengths and limitations

The main strength of this study is the large sample representing a cross-sectional population sample of 10th grade student in Norway. However, due to the observational design of the study and the self-reported data, the outcomes might have been subject to recall or social desirability bias [34]. Despite the large number of adolescents included in the study, several adolescents who had filled in implausible answers on how much they slept were removed from the analysis. There is certain risk that those giving implausible response also have different sleeping and drinking patterns than the rest, in other words, this could have led to biased effect measured estimates. In addition, we did not use a standardized sleep questionnaire which would have enabled us to investigate other potential symptoms of reduced sleep. There is also the possibility that those who were not at school at the day of the questionnaire completion or whose parents did not consent differed from those who were present. For example, they could have comprised of a group of adolescents with less healthy lifestyle, lower socioeconomic status which could have affected our effect estimates since previous research has showed that higher educated parents tend to give consent more often [35]. Therefore resulting in a possible selection bias.

Another limitation to the study was that we were not able to adjust sufficiently for possible confounders such as estimates of parental-adolescent relations, attitudes towards healthy lifestyle, mental health, other caffeine sources and other socio-economic status measurements. In other words, causality cannot be established with this type of study design. Finally, since we did not investigate other potential sources of caffeine, such as coffee and tea, the impact of ED consumption on sleep might be over-estimated. On the other hand, we believe that these are valid results as previous studies have showed that adolescents do not drink much coffee and tea at this specific age [36].

Future research should consider measuring the specific metabolic effects of ED in adolescents, next to a longer follow-up period to better understand of the potential long-term effects of ED consumption.

## Conclusion

There is an association between ED consumption and both sleep duration and shuteye latency, where increasing frequency of ED consumption is associated with longer shuteye latency and shorter sleep duration. The dose-response relationship between ED consumption and sleep suggests that there is a need for more awareness of the consequences of ED consumption among adolescents next to more information on the potential long-term effects of ED consumption and reduced sleep opportunities.

## Supplementary Information

**Additional file 1: Figure S1.** Flowchart.

## Data Availability

The datasets analysed during the current study are not publicly available due to national ethical standards, but are available from the corresponding author on reasonable request.

## Abbreviations

ED: Energy drinks
RD: Risk Difference
RR: Relative Risk

## References

[1] Dewald JF, Meijer AM, Oort FJ, Kerkhof GA, Bögels SM (2010). The influence of sleep quality, sleep duration and sleepiness on school performance in children and adolescents: a meta-analytic review. Sleep Med Rev.

[2] Hysing M, Harvey AG, Linton SJ, Askeland KG, Sivertsen B (2016). Sleep and academic performance in later adolescence: results from a large population-based study. J Sleep Res.

[3] Hirshkowitz M, Whiton K, Albert SM, Alessi C, Bruni O, DonCarlos L (2015). National Sleep Foundation's sleep time duration recommendations: methodology and results summary. Sleep Health.

[4] Saxvig IW, Bjorvatn B, Hysing M, Sivertsen B, Gradisar M, Pallesen S (2020). Sleep in older adolescents. Results from a large cross-sectional, population-based study. J Sleep Res.

[5] Woods HC, Scott H (2016). #Sleepyteens: social media use in adolescence is associated with poor sleep quality, anxiety, depression and low self-esteem. J Adolesc.

[6] Hale L, Guan S (2015). Screen time and sleep among school-aged children and adolescents: a systematic literature review. Sleep Med Rev.

[7] Orchard F, Gregory AM, Gradisar M, Reynolds S (2020). Self-reported sleep patterns and quality amongst adolescents: cross-sectional and prospective associations with anxiety and depression. J Child Psychol Psychiatry.

[8] Gillis BT, El-Sheikh M (2019). Sleep and adjustment in adolescence: physical activity as a moderator of risk. Sleep Health.

[9] Brand S, Gerber M, Beck J, Hatzinger M, Pühse U, Holsboer-Trachsler E (2010). High exercise levels are related to favorable sleep patterns and psychological functioning in adolescents: a comparison of athletes and controls. J Adolesc Health.

[10] Aepli A, Kurth S, Tesler N, Jenni OG, Huber R (2015). Caffeine consuming children and adolescents show altered sleep behavior and deep sleep. Brain Sci.

[11] VKM, Bruzell E, Carlsen MH, et al. Risk assessment of energy drinks and caffeine. Scientific opinion of the Panel of Food Additives, Flavourings, Processing Aids, Materials in Contact with Food, and Cosmetics of the Norwegian Scientific Committee for Food and Environment. Oslo: Norwegian Scientific Committee for Food and Environment (VKM); 2019. Report No.: 01.

[12] EFSA Panel on Dietetic Products N, Allergies (2015). Scientific opinion on the safety of caffeine. EFSA J.

[13] Mathew GM, Reichenberger DA, Master L, Buxton OM, Chang A-M, Hale L (2022). Too jittery to sleep? Temporal associations of Actigraphic sleep and caffeine in adolescents. Nutrients..

[14] Lebacq T, Desnouck V, Dujeu M, Holmberg E, Pedroni C, Castetbon K (2020). Determinants of energy drink consumption in adolescents: identification of sex-specific patterns. Public Health.

[15] Koivusilta L, Kuoppamäki H, Rimpelä A (2016). Energy drink consumption, health complaints and late bedtime among young adolescents. Int J Public Health.

[16] Zucconi S, Volpato C, Adinolfi F, et al. "Gathering consumption data on specific consumer groups of energy drinks". Supporting Publications; 2013. p. EN–394:190. Available online: www.efsa.europa.eu/publications.

[17] Larson N, DeWolfe J, Story M (2014). Adolescent consumption of sports and energy drinks: linkages to higher physical activity, unhealthy beverage patterns, cigarette smoking, and screen media use. J Nutr Educ Behav.

[18] Richards G, Smith AP (2016). Breakfast and energy drink consumption in secondary school children: breakfast omission, in isolation or in combination with frequent energy drink use, is associated with stress, anxiety, and depression cross-Sectionally, but not at 6-month follow-up. Front Psychol.

[19] Nowak D, Jasionowski A (2015). Analysis of the consumption of caffeinated energy drinks among polish adolescents. Int J Environ Res Public Health.

[20] Degirmenci N, Fossum IN, Strand TA (2018). Consumption of energy drinks among adolescents in Norway: a cross-sectional study. BMC Public Health.

[21] Sampasa-Kanyinga H, Hamilton HA, Chaput JP (2018). Sleep duration and consumption of sugar-sweetened beverages and energy drinks among adolescents. Nutrition..

[22] Kaldenbach S, Strand TA, Solvik BS, Holten-Andersen M (2021). Social determinants and changes in energy drink consumption among adolescents in Norway, 2017-2019: a cross-sectional study. BMJ Open.

[23] Bjertnaes AA, Grundt JH, Juliusson PB, Markestad TJ, Strand TA, Holten-Andersen MN (2019). Sex-related change in BMI of 15- to 16-year-old Norwegian girls in cross-sectional studies in 2002 and 2017. BMC Pediatr.

[24] Bjertnaes AA, Schwinger C, Juliusson PB, Strand TA, Holten-Andersen MN, Bakken KS (2020). Health-Related Behaviors in Adolescents Mediate the Association between Subjective Social Status and Body Mass Index. Int J Environ Res Public Health.

[25] Association WM (2013). World medical Association declaration of Helsinki: ethical principles for medical research involving human subjects. JAMA..

[26] Health Research Act (Helseforskningsloven) 2008. Available from: https://lovdata.no/dokument/NL/lov/2008-06-20-44. [2022 January 28th]

[27] StataCorp. (2019). Stata statistical software: release 16.

[28] Park S, Lee Y, Lee JH (2016). Association between energy drink intake, sleep, stress, and suicidality in Korean adolescents: energy drink use in isolation or in combination with junk food consumption. Nutr J.

[29] Visram S, Cheetham M, Riby DM (2016). Consumption of energy drinks by children and young people: a rapid review examining evidence of physical effects and consumer attitudes. BMJ Open.

[30] Buchanan L, Yeatman H, Kelly B, Kariippanon K (2018). Digital promotion of energy drinks to young adults is more strongly linked to consumption than other media. J Nutr Educ Behav.

[31] Huhtinen H, Lindfors P, Rimpelä A. Adolescents’ use of energy drinks and caffeine induced health complaints in Finland: Arja Rimpelä. Eur J Pub Health. 2013;23(suppl_1):ckt123.050.

[32] Stormark KM, Fosse HE, Pallesen S, Hysing M (2019). The association between sleep problems and academic performance in primary school-aged children: findings from a Norwegian longitudinal population-based study. PLoS One.

[33] Nelson MC, Story M, Larson NI, Neumark-Sztainer D, Lytle LA (2008). Emerging adulthood and college-aged youth: an overlooked age for weight-related behavior change. Obesity (Silver Spring).

[34] Livingstone MB, Robson PJ, Wallace JM (2004). Issues in dietary intake assessment of children and adolescents. Br J Nutr.

[35] Tigges BB (2003). Parental consent and adolescent risk behavior research. J Nurs Scholarsh.

[36] Forbrukerrådet (2018). Energidrikk, barn og unge.

