# Supplementary figures and images for: Sleep and energy drink consumption among Norwegian adolescents – a cross-sectional study
Source: BMC Public Health. 2022 Mar 18;22:534. doi: 10.1186/s12889-022-12972-w (PMC8932303; doi:10.1186/s12889-022-12972-w)

Figure S1: Flowchart

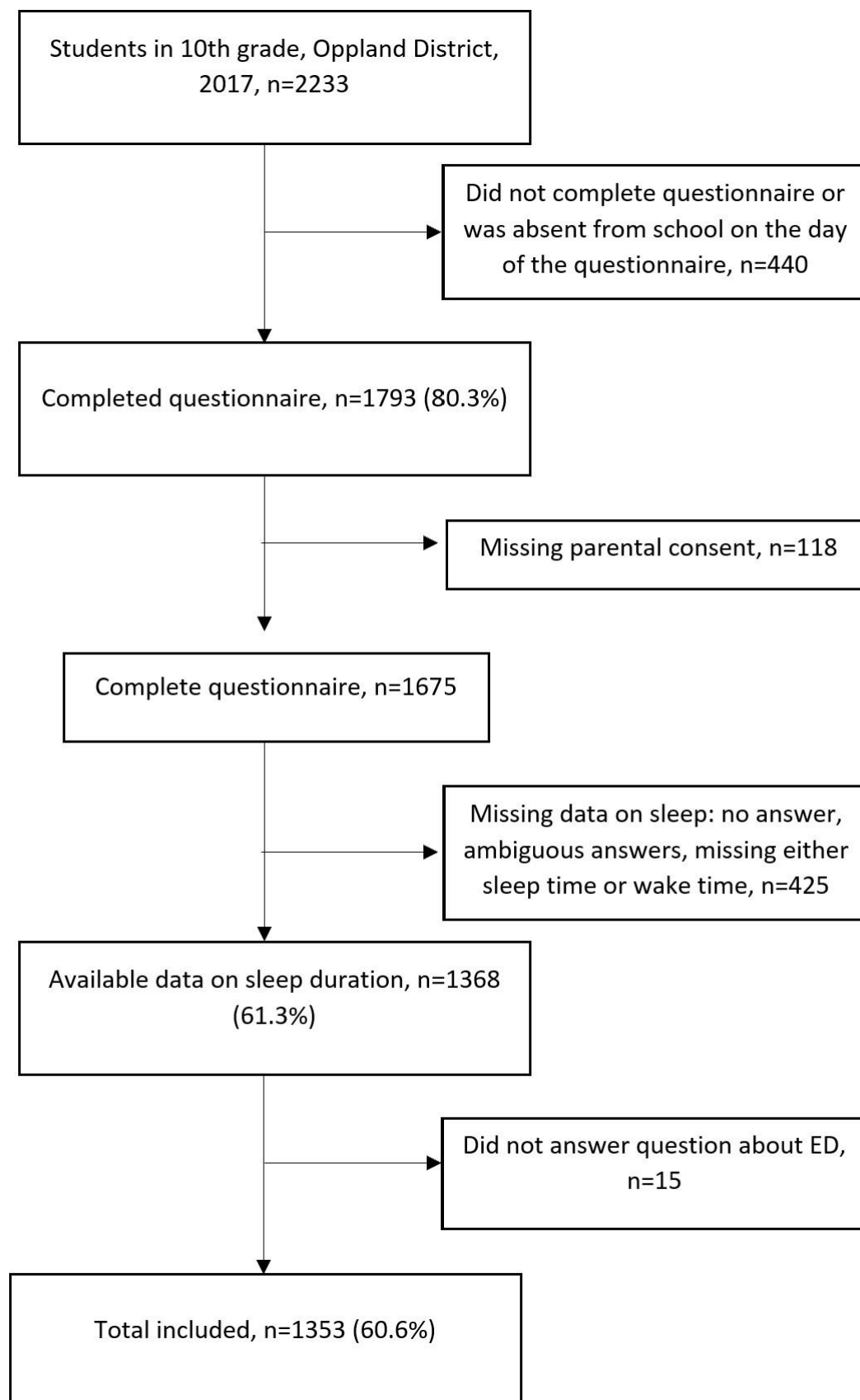

Supplement: Supplementary file 1 — Additional file 1: Figure S1. Flowchart. [file 12889_2022_12972_MOESM1_ESM.pdf]
